# Rheology of Dental Photopolymers for SLA/DLP/MSLA 3D Printing

**DOI:** 10.3390/polym17192706

**Published:** 2025-10-08

**Authors:** Luka Šimunović, Luka Brenko, Antun Jakob Marić, Senka Meštrović, Tatjana Haramina

**Affiliations:** 1 Department of Orthodontics, School of Dental Medicine, University of Zagreb, 10000 Zagreb, Croatia; mestrovic@sfzg.unizg.hr; 2 Department of Materials, Faculty of Mechanical Engineering and Naval Architecture, University of Zagreb, 10000 Zagreb, Croatia; luka.brenko@fsb.unizg.hr; 3 Faculty of Mechanical Engineering and Naval Architecture, University of Zagreb, 10000 Zagreb, Croatia; antun@3dtech.hr

**Keywords:** rheology, dental 3D printing, photopolymer resins

## Abstract

Vat photopolymerization 3D printing, including stereolithography (SLA), digital light processing (DLP), and masked SLA (mSLA), has transformed dental device fabrication by enabling precise and customizable components. However, the rheological behavior of photopolymer resins is a critical factor that governs the printability, accuracy, and performance of printed parts. This review surveys the role of viscosity, shear-thinning, and thixotropy in defining the “printability window” of dental resins and explores the relationship between these properties and the formulation and final material performance. Rheological characterization using rotational rheometry provides key insights, with shear rate sweeps and thixotropy tests quantifying whether a resin behaves as Newtonian or pseudoplastic. The literature shows that optimal printability typically requires resins with low to moderate viscosity at shear, moderate thixotropy for stability, and formulations balanced between high-strength oligomers and low-viscosity diluents. The addition of fillers modifies the viscosity and dispersion, which can improve reinforcement but may reduce print resolution if not optimized. Thermal and optical considerations are also coupled with rheology, affecting the curing depth and accuracy. In conclusion, controlling resin rheology is essential for bridging material formulation with reliable clinical outcomes, guiding both resin design and printer process optimization in modern dental applications.

## 1. Introduction

Vat photopolymerization 3D printing—including stereolithography (SLA), digital light processing (DLP), and masked SLA (mSLA)—has transformed dental device fabrication by enabling the customized, high-precision production of surgical guides, models, splints, crowns, and orthodontic aligners in biocompatible resins [1,2,3,4]. While optical and curing considerations have traditionally received more attention, an equally critical but often underappreciated factor is resin rheology which refers to the process-relevant flow and deformation behavior of the uncured liquid. Rheology governs how resins recoat, stabilize within the vat, and resist or yield under peel forces, ultimately determining whether a formulation can be printed reliably and whether the final part achieves the intended accuracy and performance [5,6].

In vat photopolymerization, the rheological behavior of dental resins must be interpreted under the shear and time scales of printing—recoating, separation, and layer resting. Four descriptors are particularly decisive: viscosity at relevant shear rates and temperatures, shear-thinning index (n), thixotropic recovery, and yield stress. These serve as practical predictors of printability, rather than generic textbook parameters. Viscosities are typically reported as ~0.1–1.5 Pa·s (γ̇ ≈ 10–50 s^−1^, 25–30 °C) for DLP/MSLA to enable self-leveling within seconds, and up to ~3 Pa·s for SLA, although the exact limits depend on printer hardware and recoating mechanisms [7]. A shear-thinning index of 0.5–0.9 is commonly reported for printable resins [8], which lowers η during recoating but restores stability at rest. Thixotropy must be strong enough to prevent the sedimentation of fillers or pigments over several hours but weak enough to recover the flow within a single layer cycle (<10 s recovery time is often cited) [8]. Although the yield stress is useful for resisting dripping, it must remain below the threshold that prevents complete gap refill [9]. These quantitative descriptors are thus not abstract but process-critical, determining whether a resin lies inside or outside the ‘printability window’.

Most commercial dental photopolymers are formulated as viscous liquids tuned through oligomer–diluent ratios, nanosilica networks, and associative thickeners to achieve a balance between fluidity and structural stability [10,11,12,13,14,15]. These microstructural elements impart shear-thinning and thixotropy, directly linking the resin composition to printability and final part precision. Although viscoelastic parameters, such as the storage and loss moduli, can also be measured, the vat photopolymerization performance is dominated by the liquid-state flow behavior. Thus, resin rheology is not an ancillary property but a key design variable that determines the recoating speed, pigment/filler suspension, dimensional fidelity, and overall process reliability [16,17]. This review is structured around three themes central to dental vat photopolymerization:

(i) Technology-specific printability windows that define the viscosity ranges, shear-thinning requirements, and thixotropic behavior for SLA, DLP, and mSLA;
(ii) Formulation strategies that shift η(γ̇,T) while preserving optical and mechanical performance; and
(iii) Opto-rheological coupling (e.g., penetration depth D_p_, scattering behavior) and its implications for processing. By bridging the fundamentals of material science with dental applications, this review provides a comprehensive framework to guide resin formulation, printer operation, and clinical translation.

## 2. Printability Window

Every 3D-printing resin has a “printability window,” a range of rheological properties within which the resin can be reliably used in vat-based printers (Table 1). A primary factor is viscosity: if it is too high, the resin may not level or reflow between layers, and if it is too low, it may drip or spread excessively. In practice, conventional bottom-up SLA/DLP systems perform best with resin viscosities up to ~2000–3000 mPa·s under optimized conditions, often with mild heating or longer settle times, although most commercial dental resins are kept in the lower hundreds of mPa·s for reliability [18,19,20]. Above this, recoating becomes unreliable—the resin does not form a uniform thin layer quickly, leading to incomplete curing or defects. Indeed, experiments have found that traditional SLA machines struggle above a few thousand mPa·s on many conventional systems unless heated or slowed; specialized linear-scan VPP exceeds this by orders of magnitude [21]. Thus, most commercial dental resins are formulated as relatively low-viscosity liquids [22]. Within this window, shear-thinning behavior is highly desirable. A resin that is thick at rest but thins under shear ensures that it stays in place when undisturbed (preventing unintended flow or dripping), yet flows easily when a recoating force is applied [23]. For instance, during layer recoating by a wiper blade or by the platform’s movement, shear-thinning allows the resin to rapidly fill the layer gap and then, once the movement stops, the viscosity partially recovers, so the resin is not very runny. Moderate thixotropy complements this: the resin can recover some structure between layer cycles, which helps keep the previous layers coated and prevents pigment settling over the print duration [24,25,26]. However, if the viscosity of the resin is too low (approaching that of water), it may over-spread and form very thin films or leak into unintended areas, reducing accuracy and potentially causing print failures due to uncontrolled polymerization outside the desired layer geometry. Manufacturers often specify an optimal viscosity range for SLA/DLP resins; for dental model materials, this is typically in the low hundreds of mPa·s at 25 °C [27,28]. Some researchers have defined printability criteria by combining viscosity and yield stress—e.g., requiring a minimum zero-shear viscosity or yield stress so that the resin does not flow under gravity alone, yet has a low enough high-shear viscosity for easy recoating [23]. Overall, the printability window is a compromise: the resin must be fluid enough to recoat 50–100 μm layers uniformly within seconds, but viscous enough at rest to maintain layer integrity. This window is crucial for achieving consistent layer thickness and preventing flaws, which directly impact the accuracy and success rate of printed dental appliances.

### Technology-Specific Windows

Although SLA, DLP, and MSLA are often grouped as “vat photopolymerization,” their layer-formation mechanics impose distinct rheological requirements. Laser-scanning SLA (often with a wiper or recoater) can tolerate somewhat higher viscosities or mild thixotropy because of the mechanical action that redistributes the resin. In contrast, bottom-up DLP and MSLA expose an entire layer simultaneously and rely on rapid self-leveling and low separation forces, favoring lower viscosity and a rapid thixotropic breakdown. Table 2 summarizes the practical windows compiled from peer-reviewed studies, technical documentation, and common dental printer practices.

## 3. Methodology of Measurement

The rheological characterization of dental 3D-printing resins is essential to quantify viscosity profiles and ensure that a formulation lies within the printable window. Typically, a rotational rheometer with a cone-plate or parallel-plate geometry is used to measure the viscosity as a function of the shear rate. For example, viscosity may be measured by sweeping shear rates from 1 s^−1^ up to 100 s^−1^ at a controlled temperature (often 23–25 °C, simulating room-temperature printing) [22]. From the resulting flow curve, one can determine whether the resin is Newtonian (flat viscosity vs. shear rate) or shear-thinning (viscosity decreases with shear). The degree of shear-thinning can be quantified by fitting to a power-law model, $\eta =K{\gamma ˙}^{n-1}$, where n < 1 indicates pseudoplastic behavior (the farther n is below 1, the stronger the shear-thinning) [23,27]. Rheometers also facilitate thixotropy tests—such as applying a high shear for a set time and then observing viscosity recovery, or performing up-and-down shear rate loops to measure hysteresis. Such tests reveal how quickly a resin rebuilds its internal structure after shear, which correlates with its behavior between layer recoating steps. Besides steady shear tests, oscillatory rheometry can probe viscoelastic properties: by applying small-amplitude oscillations, to measure the storage modulus G’ (elastic response) and loss modulus G’’ (viscous response) of the uncured resin. A resin with significant G’ at rest may possess a weak gel structure (e.g., due to nanoparticles or hydrogen bonding), signifying a yield stress—a threshold stress needed to initiate flow. The yield stress can also be estimated by extrapolating the flow curves or via creep tests. A high yield stress in a resin can prevent it from leveling properly, but a slight yield stress (or high zero-shear viscosity) is often useful for the stability of suspensions [19].

For thermal effects, viscosity is usually measured at various temperatures to determine how pre-heating the resin might improve flow [39]. Dental SLA resins often exhibit an exponential decrease in viscosity with moderate heating (e.g., 50–60 °C) due to reduced monomer viscosity, which can be quantified using temperature ramp rheometry [40]. Alongside rheology, complementary methods such as differential scanning calorimetry (DSC) and real-time Fourier-transform infrared spectroscopy (FTIR) are used to assess cure kinetics (which can indirectly relate to rheology, as highly viscous formulations might hinder reactive diffusion) [41]. Finally, after printing and curing, dynamic mechanical analysis (DMA) is employed on the solid polymer to measure properties such as glass transition temperature (Tg) and modulus [27,42,43]. While DMA/DSC pertain to the cured polymer, correlating these results with the resin’s initial rheology provides insights—for example, a resin with higher initial viscosity often indicates higher oligomer content, which may translate to a higher crosslink density and Tg in the cured state [44,45]. By using a combination of rheometry (for the liquid resin) and thermal/mechanical analysis (for the cured parts), researchers can draw connections between formulation, print processability, and final part performance. Consistent methodologies and standardized rheological metrics (e.g., viscosity at 10 s^−1^, thixotropic index, and yield stress value) are important for comparing different dental resins and informing print parameter optimizations. Recommended practices and reporting standards for these measurements are summarized in Table 3.

## 4. Formulation–Rheology

**Figure 1:**
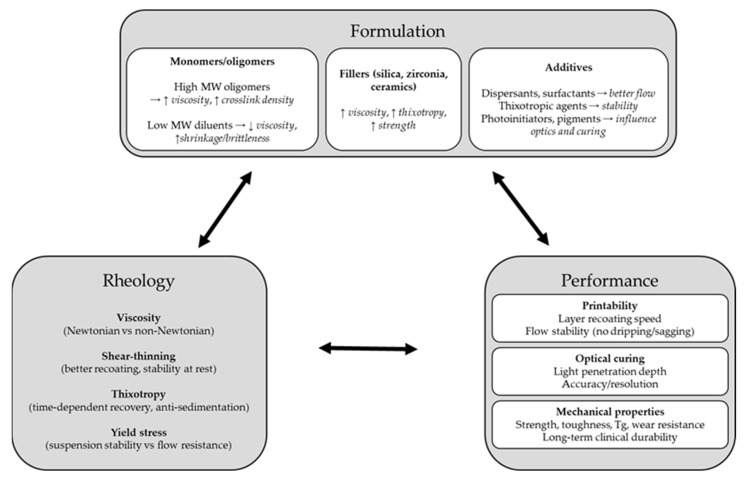
Iterative relationship of formulation, rheology, and performance in dental 3D printing resins.

The rheological profile of a photopolymer resin is fundamentally determined by its formulation, monomer/oligomer chemistry, presence of fillers, and rheological additives [19]. Dental 3D-printing resins typically contain one or more multifunctional acrylic monomers or oligomers as the base matrix. These base components can vary widely in viscosity: for instance, a high-molecular-weight urethane diacrylate or bis-GMA analog can be extremely viscous, whereas a low-molecular-weight diluent monomer such as TEGDMA or PEGDA is thin, almost water-like in viscosity [46,47]. Thus, a common strategy is to blend high-strength but viscous oligomers with low-viscosity diluents to achieve a printable consistency [21]. This is a direct trade-off: the undiluted oligomer might yield superior mechanical properties in the cured state, but its viscosity could be excessively high. By diluting it, the viscosity can be brought into the hundreds of mPa·s, at the cost of introducing more reactive diluent into the network [48,49]. However, excessive use of diluents is detrimental, as it can lead to increased polymerization shrinkage and brittleness, and reduce the final cross-link density or Tg of the polymer [50]. As one study noted, reactive diluents (e.g., triethylene glycol dimethacrylate, trimethylolpropane triacrylate) are effective at lowering resin viscosity and improving print flow, but using too high a fraction “may deteriorate the mechanical properties of the resin” after curing [51]. The iterative relationship between formulation, rheology, and printed-part performance is illustrated in Figure 1, highlighting how changes in composition directly influence flow behavior and final material properties. Practically, for bottom-up DLP/MSLA, a target viscosity of 0.1–1.5 Pa·s at 25–30 °C (γ̇ ≈ 10–50 s^−1^) balances rapid self-leveling with manageable peel forces; SLA with a recoater can tolerate up to ≈3 Pa·s given longer settling times or heating. When η exceeds ≈2–3 Pa·s under operating conditions, incomplete gap refill and elevated separation forces become likely unless cycle times are increased or the vat is heated [28,52].

Beyond monomers, fillers and additives significantly influence rheology. Many dental resins (especially those for models or permanent restorations) incorporate reinforcing fillers like silica, zirconia, or other ceramics at small particle sizes [53,54]. These particles increase the viscosity in two ways: by the volume fraction effect (more solids to move in the liquid) and by creating a particle network (particularly if nano-silica or similar is used, which can form a percolating network via hydrogen bonding or van der Waals forces) [55]. Even a few weight percent of fumed silica, for example, can turn a low-viscosity resin into a gel-like thixotropic fluid [56]. One report demonstrated that adding hydrophilic fumed silica significantly increased the zero-shear viscosity and imparted strong thixotropy to a UV-curable ink, whereas a hydrophobic silica had a lesser effect [57]. Fillers often cause the resin’s flow shear-thinning: the agitation breaks down particle agglomerates or disrupts temporary networks, lowering viscosity under shear. Indeed, adding rigid fillers or fibers to a previously Newtonian photopolymer typically converts its behavior to pseudoplastic [58]. This is beneficial for printing (as discussed, shear-thinning aids recoating), but if the viscosity at low shear becomes too high, it can be problematic for the printer’s recoating mechanics [59]. To counter this, dispersants or surfactants are frequently added to stabilize particle distribution and moderate the thickening. For instance, in highly filled ceramic–resin hybrids, comb-like dispersant molecules can adsorb on particle surfaces, preventing agglomeration and reducing viscosity [60]. Rheological modifiers such as polyhedral oligomeric silsesquioxanes (POSS) or certain thixotropic agents can be added in small amounts to fine-tune flow—either to increase viscosity for stability or to induce shear-thinning behavior [61]. However, every additive can affect the cure chemistry (e.g., inhibiting polymerization if not chosen carefully) and the optical properties; therefore, formulation changes require holistic evaluation. A concise summary of formulation levers and their expected rheological or optical outcomes is provided in Table 4 which offers practical guidance for balancing viscosity, printability, and optical clarity. In summary, rheology is a direct reflection of formulation: high-viscosity base resins demand diluents; fillers demand dispersants; and any target rheological profile (such as slight thixotropy) must be achieved by choosing the appropriate ingredients. Dental resin development is thus an exercise in multi-variable optimization, ensuring that the combination of monomers, fillers, photoinitiators, and additives yields a resin that prints reliably and leads to a strong, accurate dental appliance. Formulators extensively leverage rheological models and measurements during this process to predict how a given formulation adjustment will affect viscosity curves and printability.

## 5. Rheology–Optics

Interestingly, the flow properties of a resin are interlinked with its optical behavior during printing. Vat photopolymerization relies on UV or visible light penetrating through the liquid resin to cure specified regions. If a resin’s formulation (and thus its rheology) includes components that affect optical transmission—such as pigments, dyes, or solid fillers—this can alter the cure depth and accuracy of printing. Highly filled resins, which as noted tend to be more viscous, often scatter and absorb more light, reducing the penetration depth of the curing radiation. In dental contexts, for example, adding TiO_2_ or other opacifiers to match the tooth color will increase viscosity but also limit how far light can cure the material, requiring thinner layers or higher exposure doses. Researchers have emphasized that the cure kinetics and resolution in SLA are influenced not only by printer settings but also by resin properties like viscosity and the presence of light-absorbing additives [63,64]. A viscous resin with high pigment content might cure only a shallow layer per exposure because the light intensity decays faster compared to a clear, low-viscosity resin. This “optical printability” window must overlap with the rheological printability window: if we raise filler or dye content to improve mechanical or esthetic properties (raising viscosity in the process), we might compromise optical curing efficiency. The key interactions between rheological parameters and optical performance are summarized in Table 5. For instance, one study of a nanoparticle-filled SLA resin found that beyond a certain filler loading, the scattering impeded curing so much that the effective cured layer thickness dropped, defeating the purpose of adding more filler for strength [65]. Thixotropy and clarity are also related—some thixotropic agents (like fumed silica) can increase haze in the resin, affecting the precision of projected images in DLP systems [57]. The particle dispersion quality (which is a rheological concern) is therefore critical to optical homogeneity; agglomerates not only thicken the resin but can act as micrometer-scale blockers or scatterers of light, causing uneven curing.

Another aspect is that rheology impacts how the resin flows around already cured features during printing, which can influence the light exposure of those features. In bottom-up printing, after a layer is cured and the platform lifts, the resin flows in to cover the next layer. If the resin is too viscous, it might not fully refill gaps or might trap air bubbles, resulting in areas where the next UV exposure passes through uneven resin thickness or voids. This can lead to dimensional inaccuracies or cure inhibition in those spots. A suitably low viscosity (or using measures like gentle resin stirring between layers) ensures consistent optical paths for each layer exposure. Conversely, a resin that has extremely low viscosity might run off vertical surfaces quickly, meaning that freshly cured layers could be less supported by surrounding liquid, potentially leading to cure-through or light bleed in unintended areas. In practice, manufacturers sometimes add a slight pigment (like a yellow or orange dye) to limit the cure depth and improve z-resolution; these dyes typically have a minimal effect on viscosity at the low concentrations used. However, any solid UV blockers (e.g., ceramic particles) will affect rheology. Thus, developing a resin for, say, a color-matched 3D printed tooth requires balancing the filler-induced viscosity increase against the need for sufficient light penetration. Advanced formulations use refractive index matched fillers (to minimize scattering) and highly efficient photoinitiators to mitigate optical issues while still benefiting from the filler’s mechanical contribution [66]. In summary, the rheological and optical properties of dental 3D printing resins are coupled through the formulation ingredients: achieving high precision and reliable curing demands a resin that not only flows well but also transmits and interacts with light in a controlled manner. Multidisciplinary evaluations—rheometry for flow and spectrophotometry or cure tests for optics—are therefore key in qualifying a new dental resin.

### Quantitative Coupling Between Particle Size, Scattering, and Cure Depth

The working curve C_d_ = D_p_ln(E/E_c_) links the cure depth C_d_ to penetration depth D_p_ and critical exposure E_c_ [67]. Fillers modify D_p_ via absorption and scattering; for sub-wavelength particles, Rayleigh scattering scales as ∝r^6^/λ^4^, while for particles comparable to λ, Mie scattering dominates and tends toward forward scattering [65]. Empirically, nano-scale fillers reduce D_p_ more steeply than equal-volume micro-fillers due to the larger number density of scattering centers; simultaneously, nano-fillers raise viscosity more per wt% than micro-fillers [68]. Consequently, for dental resins requiring thin layers (25–100 µm), we recommend the following:

(i) Index-match filler to matrix (|Δn| → 0) to suppress scattering and preserve D_p_ [69];
(ii) Favor sub-micron (~0.1–1 µm) over deep-nano sizes when high loadings are needed, to minimize number density while retaining smooth surfaces [68];
(iii) Calibrate D_p_ after any filler or dye change and target D_p_ ≈ 1.1–1.5 × layer height to ensure Z-bond without overcure [67,70].

## 6. Rheology–Mechanical Properties

The ultimate mechanical properties of a 3D-printed dental object (such as an aligner, splint, or crown) stem from the polymer network formed during curing—and this network is influenced by the resin’s rheology and composition in the uncured state. There are several connections between resin rheology and mechanical outcomes:

### 6.1. Monomer Functionality and Crosslink Density

More viscous resin formulations usually contain higher fractions of large, multi-functional oligomers. These oligomers (e.g., polyurethane acrylates or bis-EMA) form highly crosslinked networks upon polymerization, typically yielding higher stiffness and strength in the cured part. In contrast, very fluid resins have more low-viscosity diluents, which often act as flexibilizers or chain extenders in the polymer matrix, leading to lower modulus and strength. In other words, to obtain a tough, strong dental appliance, one leans toward a more viscous formulation—but that must be balanced with printability [21]. A recent study highlighted that when high-performance acrylate oligomers were diluted to reduce viscosity (down to ~5000 mPa·s) for printing, the original mechanical properties were compromised relative to the neat oligomer [21]. Similarly, a recent review pointed out that while adding reactive diluents improves printability, “excessive use may deteriorate mechanical properties” like flexural strength and hardness [52]. These findings underscore that mechanical performance (hardness, tensile/flexural strength, fracture toughness) is highest when the resin has a robust crosslinked network—which correlates with a formulation that, before curing, was on the higher end of viscosity (rich in network-forming components).

### 6.2. Filler Content

Adding fillers (which increases viscosity and often induces shear-thinning) generally boosts the mechanical properties of the cured composite, up to a point. For example, incorporating a nano-ceramic like zirconia or silica can raise the stiffness, wear resistance, and fracture toughness of a printed dental resin. One case study showed that mixing a small amount of potassium titanate ceramic into a base resin substantially increased its flexural strength (from ~15 MPa to ~30–39 MPa range, depending on loading) [52]. The same composite saw improvements in Shore D hardness and tensile strength as well. However, these gains were only realized when the filler was well-dispersed, which the authors achieved by adding a bit of PEG as a dispersing aid [52]. The PEG helped control the viscosity increase and allowed higher resolution printing (thinner line patterns) than a resin with poorly dispersed filler [52]. Thus, rheology management (through additives) was integral to obtaining the mechanical benefits of the filler. If the filler had formed clumps due to inadequate dispersion (leading to very high local viscosity), the printed parts would have defects and likely poorer mechanical behavior. Well-balanced rheology ensures uniform particle distribution, translating to consistent reinforcement throughout the polymer matrix.

### 6.3. Degree of Cure and Network Formation

Rheology also connects to how thoroughly a resin cures, which in turn affects mechanical properties. A resin that is too viscous might inhibit complete mixing of photoinitiator or oxygen scavengers, or it might not flow into fine feature details, causing microscopic uncured regions. Conversely, printing under conditions that lower viscosity—such as elevated temperature—can improve cure. An in vitro study on dental resins found that printing at 70 °C (as opposed to room temperature) significantly increased the double-bond conversion (from about 67% to 86%) and produced specimens with notably better mechanical properties [40]. Heating reduced the resin viscosity during printing, facilitating more complete polymerization and crosslinking in each layer, and the post-cured hardness and strength were higher as a result [40]. This example illustrates that process-induced rheological changes (like viscosity reduction by heat or by mixing) can lead to a more tightly crosslinked polymer network, thereby improving mechanical outcomes. On the flip side, if a resin has high thixotropy or yield stress, there is a possibility of trapping bubbles or causing uneven curing in internal regions, which would act as flaws and weaken the part. Hence, achieving a mechanically robust printed piece requires ensuring the resin flows into all geometrical features and cures uniformly. Rheology is central to both of those requirements.

In summary, a direct correlation exists: formulation choices that enhance mechanical performance (high-functionality monomers, added reinforcements) tend to raise viscosity, making printing harder. The resin developer’s task is to use tools (diluents, dispersants, rheology modifiers) to bring viscosity into a printable range without erasing the mechanical advantages. By monitoring rheological parameters and mechanical test results in tandem, one can identify the sweet spot—for instance, a resin that is just fluid enough to print at 25 °C, but as concentrated as possible in structural building blocks to yield a stiff, strong product. Modern dental materials research often iterates formulations with this in mind, measuring the viscosity and shear-thinning index for each tweak and then measuring the DMA or flexural strength of printed samples. The interplay of rheology and final properties is complex, but mastering it is key to producing next-generation 3D-printed dental materials that are both easy to print and strong in service.The main rheology–mechanical trade-offs are summarized in Table 6.

## 7. Discussion

From the above, it is evident that rheology sits at the nexus of printability and performance for dental photopolymers. Optimizing a resin’s rheological profile is therefore a primary consideration in product development. One recommendation is for resin manufacturers and researchers to clearly report rheological parameters (viscosity vs. shear rate curves, thixotropy index, etc.) in addition to standard mechanical properties, as this data can guide end-users in setting printer parameters (for example, knowing a resin’s viscosity can inform whether a heated vat or slower recoating might be needed). For dental clinics or labs using these materials, an awareness of how temperature affects resin viscosity is useful—simply warming the resin to 30–35 °C can significantly lower viscosity and improve layer spreading, as long as the resin’s chemistry tolerates it [40]. Printers with built-in vat heaters or resin agitation systems are recommended for higher-viscosity bio-based or filled resins, to widen the printability window. Another practical recommendation is to implement gentle mixing or oscillation of the resin between layers for viscous or thixotropic resins. Research in non-Newtonian fluids shows that applying small vibrations can reduce apparent viscosity and accelerate leveling, which could be an elegant solution to keep highly filled dental resins well-leveled without manually slowing down the print. Some high-end vat printers already use a wiper blade or resin circulation system—ensuring these are utilized (or retrofitted) can dramatically improve outcomes with “difficult” resins.

From a formulation standpoint, we recommend an integrated design approach: materials scientists should collaborate with dental experts to define the acceptable mechanical property targets and then work backwards to formulate a resin that meets those targets while remaining printable. This might involve using novel oligomers that are mid-viscosity but still impart toughness, or using nanoparticles that are surface-treated to minimize viscosity increase. The use of refractive-index matched nano-fillers is encouraged, as it reduces light scattering (so one can add more filler without requiring excessive photoinitiator or longer exposures), indirectly allowing a higher solids content (and thus higher viscosity permissible) without losing print fidelity. For very high viscosity formulations that would normally be unprintable, emerging technologies offer hope: for instance, a recent vat system employing moving rollers demonstrated the ability to print resins over 600,000 mPa·s in viscosity—a regime utterly beyond conventional SLA [21]. While not yet common, such innovations suggest that the “ceiling” of printable viscosity can be raised with clever engineering. Thus, one can envision in the near future printing dental devices from highly filled, paste-like resins that yield extremely high-strength, highly filled polymer composites, by using printers equipped with advanced recoating mechanisms or continuous liquid interfaces.

In routine practice, however, a conservative approach is warranted: stay within known good rheological ranges for the available printer. If a dental resin’s datasheet specifies 200–300 mPa·s, trying to add extra filler that doubles the viscosity is likely to cause print failures unless compensatory measures (slower print speeds, heated vats) are taken. Users should also be aware of resin aging: some photopolymers can increase in viscosity over time (due to slow polymerization or particulate settling). Regularly stirring or gently heating the resin before use can re-homogenize it, reducing viscosity variability between prints. Finally, standardizing rheological test methods in research (e.g., always reporting viscosity at a common shear rate like 50 s^−1^ and 25 °C) would greatly help compare studies. It would allow building a more quantitative map of “printability windows” for various resin classes (e.g., model resin vs. castable resin vs. biocompatible splint resin). This review has highlighted that achieving good printability does not necessarily mean compromising mechanical performance—with informed formulation (using appropriate diluents and additives) and possibly innovative hardware, one can have both. The recommendations for future work include exploring novel thixotropic agents that temporarily reduce viscosity under printer shear conditions but allow rapid solidification (almost like a Bingham plastic behavior) to see if they can improve feature resolution (especially in printing vertical walls or overhangs, where resin dripping is a concern). Additionally, in situ rheology monitoring on printers (for example, measuring the force to wipe a blade) could provide feedback to actively control print speeds or wait times, ensuring each layer is properly leveled. Such smart feedback loops would be particularly useful for resins near the edge of the printability window.

## 8. Conclusions

This review highlights the central role of rheology in dental vat-photopolymerization resin application. To ensure that the insights are directly useful for researchers and practitioners, we summarize our findings as follows:

- Design for viscosity: η (25–30 °C, γ̇ = 10–50 s^−1^) ≈ 0.1–1.5 Pa·s for DLP/MSLA, and ≤3 Pa·s for SLA with a recoater, to ensure uniform gap refill and controlled peel forces.
- Standardize reporting: Always report η at defined γ̇ and T, thixotropy recovery, τy, and E_c_/D_p_ as a minimal framework for method-to-method comparison.
- Optical tuning: Adjust the penetration depth (D_p_) with absorbers to ≈1.1–1.5× the target layer height, and re-establish E_c_ after any formulation change.
- Filler use: Employ sub-µm, index-matched fillers with validated dispersion to increase stiffness while limiting viscosity rise and scattering.
- Temperature management: Maintain the resin temperature between 25 and 30 °C to widen the printability window without altering the formulation.
- Formulation balance: Co-optimize the oligomer–diluent ratio to preserve mechanical performance (modulus, Tg) while maintaining flow and avoiding excessive dilution that causes brittleness.

## Figures and Tables

**Table 1:** Practical printability window for dental vat-photopolymerization resins.

Parameter	Typical Target/Guideline	Rationale
Viscosity at 25–30 °C (γ̇ ≈ 10–100 s^−1^)	0.1–2.0 Pa·s (100–2000 mPa·s)	Reliable self-leveling and moderate peel forces
Zero-shear viscosity/yield stress	Low-to-moderate; just enough to prevent sagging	Stability without impeding leveling; avoid trapped voids
Shear-thinning index (n in power-law)	0.5–0.9 (pseudoplastic)	Low η during recoating; higher η at rest
Operating temperature	25–30 °C (heated vat recommended)	Heating reduces viscosity; hot lithography shows improved flow and green properties
Layer height vs. optical Dp	Dp ≈ 1.1–1.5 × layer thickness	Ensures bond without overcure; aligns with Jacobs’ working curve model
Fillers (vol%)	Minimal necessary; sub-micron preferred	Balances viscosity and scattering; improves dispersion, and reduces light attenuation

References: [8,9,29,30,31,32,33,34,35].

**Table 2:** Approximate viscosity windows and rheological requirements for SLA, DLP, and MSLA vat-photopolymerization systems.

Technique	Viscosity at 25–30 °C (γ̇ ≈ 10–100 s^−1^)	Shear/Thixotropy Profile	Process Notes
SLA (laser)	~0.5–3 Pa·s typical; up to ~5 Pa·s reported for conventional SLA; >10 Pa·s only with specialized linear-scan VPP	Mild shear-thinning helpful; low-to-moderate thixotropy acceptable	Blade/wiper aids redistribution; longer settle often tolerable. Upper conventional limit ≈5000 mPa·s; ultra-high viscosity feasible only with linear-scan VPP.
DLP (proj.)	~0.1–2 Pa·s typical (100–2000 mPa·s); up to ~3 Pa·s possible with heating and very slow peel; higher viscosities generally require non-standard hardware	Shear-thinning needed; keep yield stress low enough to allow complete re-wetting	Fast self-leveling and minimized peel forces; heated vats often used. Ceramic DLP slurries printable in the ~1–2 Pa·s range at relevant shear rates.
MSLA (LCD)	~0.1–1.5 Pa·s typical, evidenced by multiple LCD/MSLA datasheets (≈0.36–0.52 Pa·s common; some flexible resins up to ~2.2 Pa·s)	Similar to DLP; very short cycles demand rapid shear-thinning and recovery	Keep resin warm; avoid heavy pigments that hinder leveling. Vendor TDS show LCD/MSLA resins commonly 300–520 mPa·s, with some formulations near ~2 Pa·s.

References [9,21,36,37,38].

**Table 3:** Recommended rheological characterization and reporting.

Item	Recommended Practice	Reported Metric
Geometry/gap	Cone-plate (1–2°) or parallel-plate; 25–30 °C	Geometry, gap, temperature
Shear sweep	γ̇ = 0.1–100 s^−1^ (both up and down)	η(γ̇) curve; power-law fit (K, n)
Thixotropy/recovery	3-interval test: low–high–low shear (e.g., 0.1/50/0.1 s^−1^)	% viscosity recovery at 30–60 s; hysteresis area
Yield stress	Stress ramp or creep tests	τy (Pa) and method
Temp dependence	20–60 °C ramp	Arrhenius fit (activation energy for flow)
Viscoelasticity	SAOS (G′, G′′, tan δ) in LVR	G′, G′′ vs. ω at 25–30 °C
Reporting	Always include temperature and shear-rate for η values	η at 10 s^−1^ and 50 s^−1^ (25 °C/30 °C)

References [8,9,30,31].

**Table 4:** Formulation levers and expected rheological/optical outcomes.

Lever	Primary Effect on Flow	Secondary Effects	Practical Guidance
↑ Oligomer (UDMA/Bis-GMA)	↑ η; mild shear-thinning	↑ modulus, Tg; ↓ shrinkage	Dilute with reactive monomer to keep η < 2 Pa·s.
Reactive diluent (TEGDMA)	↓ η; often Newtonian	↑ crosslink density; risk brittleness	Limit to minimum that achieves printability.
Nanofiller (silica, etc.)	+ thixotropy; ↑ η at low shear	↑ stiffness; ↑ scattering if mismatched	Use sub-µm; surface-treat; disperse ultrasonically.
Pigments/absorbers	~η neutral (low wt%)	Tune Dp; color/opacity	Use potent dyes at low ppm; avoid large pigments.
Index matching	—	↓ scattering; ↑ Dp at a given loading	Match n(resin) ≈ n(filler) to preserve cure depth.

References [30,34,35,62,63]; (↑ means increase and ↓ means decrease).

**Table 5:** Coupling between flow and optics.

Factor	Optical Impact	Mitigation
↑ Filler (vol%)	↓ D_p_; ↑ lateral light spread (XY overcure)	Limit loading; index-match; finer layers
Smaller particles	More centers → ↑ scattering; ↓ Dp	Use sub-µm but avoid excessive nano-loadings; disperse well
High viscosity	Non-uniform films; trapped bubbles → cure noise	Warm to 25–30 °C; add settle time; mild agitation
Thixotropy (high)	Incomplete re-wetting; uneven exposure	Keep τy low; ensure rapid breakdown under shear
D_p_ vs. layer	Dp too low → poor Z-bond; too high → z-bleed	Tune absorber to D_p_ ≈ 1.1–1.5 × layer thickness

References: [9,28,30,31,32,33,34,35,62]; (↑ means increase and ↓ means decrease).

**Table 6:** Rheology—mechanical trade-offs.

Change	Typical Mechanical Outcome	Printability Implication
↑ Oligomer fraction	↑ modulus/strength/Tg; ↓ shrinkage	↑ η; may require heating/slower cycles
+Sub-µm filler (5–15 wt%)	↑ stiffness/hardness; radiopacity	↑ η + shear-thinning; check dispersion and Dp
+Excess diluent	Easier flow; risk brittleness/shrinkage	↓ η; consider toughener; ensure full conversion
Higher print temp (30 °C)	↑ conversion; ↑ strength (via lower η during cure)	Validate dimensional accuracy; recalibrate Ec

References: [9,30,31,32,33,34,35,62]; (↑ means increase and ↓ means decrease).

## Data Availability

No new data were created or analyzed in this study. Data sharing is not applicable to this article.
